# Validity of submaximal aerobic capacity and strength tests in firefighters

**DOI:** 10.1093/occmed/kqae004

**Published:** 2024-02-21

**Authors:** W Hart, D Taylor, D C Bishop

**Affiliations:** North Yorkshire Fire and Rescue Service, Transport and Logistics Hub, Thirsk, UK; School of Sports and Exercise Science, University of Lincoln, Lincoln, UK; School of Sports and Exercise Science, University of Lincoln, Lincoln, UK

## Abstract

**Background:**

Typically, the fitness of UK firefighters is assessed via submaximal estimate methods due to the low demands on time, money, expertise and equipment. However, the firefighter-specific validity of such testing in relation to maximum aerobic capacity ($\dot{V}O_{2\max}$) and particularly muscular strength is not well established.

**Aims:**

To examine the validity of submaximal methods to estimate $\dot{V}O_{2\max}$ and maximal strength in operational firefighters.

**Methods:**

Twenty-two full-time operational firefighters (3 female) completed same-day submaximal (Chester Step Test; CST) and maximal (treadmill) assessments of $\dot{V}O_{2\max}$, with a sub-sample of 10 firefighters (1 female) also completing submaximal and maximal back-squat (i.e. one repetition maximum; 1RM) assessments. All participants then completed the Firefighter Simulation Test (FFST) within 2–4 days.

**Results:**

CST underestimated actual $\dot{V}O_{2\max}$ by 1.4 ml·kg^–1^·min^–1^ (~3%), although $\dot{V}O_{2\max}$ values were positively correlated (*r* = 0.61, *P* < 0.01) and not significantly different. Estimated $\dot{V}O_{2\max}$ values negatively correlated with FFST performance (*r* = –0.42). Predicted 1RM underestimated actual 1RM by ~2%, although these values were significantly correlated (*r* = 0.99, *P* < 0.001) and did not significantly differ. The strongest predictive model of FFST performance included age, body mass index, and direct maximal measures of 1RM and $\dot{V}O_{2\max}$.

**Conclusions:**

Submaximal back-squat testing offers good validity in estimating maximum firefighter strength without exposure to the fatigue associated with maximal methods. The CST provides a reasonably valid and cost-effective $\dot{V}O_{2\max}$ estimate which translates to firefighting task performance, although the error observed means it should be used cautiously when making operational decisions related to $\dot{V}O_{2\max}$ benchmarks.

## Teaser

Although aerobic fitness and strength are important to firefighters, the accuracy of submaximal tests to profile these during recruitment and/or annual testing is still not well established. This study examined how well submaximal stepping and weight-lifting tests estimate the maximum aerobic capacity and strength of firefighters, respectively. Estimated maximum strength demonstrated good accuracy, whilst estimates of maximum aerobic capacity were less accurate and should be used cautiously when making operational decisions relating to firefighter fitness.

**Key learning points**

**What is already known about this subject**

- Firefighting requires sufficient levels of cardiorespiratory fitness and muscular strength to meet the demands of the job, with a failure to meet minimum standards in these components leading to withdrawal from operational service.
- Annual fitness testing of firefighters is commonly performed via submaximal estimate methods due to relatively low demands on time, money, expertise and equipment.
- The firefighter-specific validity of testing in relation to aerobic capacity and particularly muscular strength is not well established, which may lead to erroneous operational decisions being made.

**What this study adds**

- The Chester Step Test provides a reasonably valid and cost-effective $\dot{V}O_{2\max}$ estimate in firefighters, although the associated error means it should be used cautiously when making operational decisions.
- A novel submaximal back-squat protocol can provide a valid estimate of lower-body maximal strength in firefighters.

**What impact this may have on practice or policy**

- If the Chester Step Test is utilized during firefighter recruitment and/or annual fitness testing then borderline results (i.e. within ±12.16 ml·kg^–1^·min^–1^ of the operational benchmark for $\dot{V}O_{2\max}$) should be confirmed on a case-by-case basis via maximal incremental testing and direct gas analysis.
- Estimating maximal strength via submaximal protocols and laser-optic device technology is a sufficiently valid, simple and cost-effective method to be considered as part of the standard approach to fitness profiling across the fire and rescue service.
- The sex- and age-specific relevance of operational benchmarks for both maximum aerobic capacity and strength should continue to be critically explored via firefighter-specific research studies, particularly in females.

## Introduction

Firefighters are expected to possess sufficient levels of cardiorespiratory fitness and muscular strength to successfully meet the demands of the occupation [1–4]. It is, therefore, standard practice for firefighters to complete annual cardiorespiratory fitness and strength tests, with a failure to meet service-specific standards deeming them unfit to perform operational duties. These are used alongside the recently developed Firefighting Simulation Test (FFST) to confirm that operational fire and rescue staff meet minimum job-specific functional requirements [5,6]. Given the implications of test outcomes to individual firefighters and the level of coverage provided by each service, the need for practitioners to have reliable and valid data to assess and develop firefighter cardiorespiratory fitness and muscular strength is essential [6].

A common method of testing cardiorespiratory fitness in UK firefighters is the submaximal Chester Step test (CST), which offers good test–retest reliability in estimating maximal aerobic capacity ($\dot{V}O_{2\max}$) [7,8] and is considered an accessible method of assessing $\dot{V}O_{2\max}$ within occupational settings such as firefighting [9,10]. However, $\dot{V}O_{2\max}$ estimates from the CST may significantly differ from values obtained via direct maximal treadmill testing [7] and there remains limited evidence regarding the validity of the CST in firefighting specifically. As such, there is a need for research that helps inform and support decisions made using the CST within the service (i.e. when profiling the fitness of active personnel).

Maximum multiple (e.g. five repetition max) or single repetition max (1RM) tests are commonly used to assess the muscular strength of firefighters, with good levels of validity reported across various upper-body 1RM tests in comparison with job-specific tasks [11,12]. Whilst lower-body muscular strength may also contribute to firefighter fitness and task performance [13,14], methods that have been validated specifically for firefighters are lacking. Furthermore, maximal strength testing may be time consuming and lead to fatigue that may place firefighters at greater risk during subsequent operational duties. Submaximal methods may help establish minimum operational standards of muscular strength in an efficient manner whilst also mitigating the risks associated with maximal test protocols [15–17]. The integration of laser-optic device technology with submaximal barbell lifting has emerged as a relatively simple and cost-effective way to provide valid and reliable estimates of maximal strength [18,19]. However, this approach has not yet been explored in relation to physically demanding occupations which rely on profiling of muscular strength, such as the UK Fire and Rescue Service.

Given these points, this study examined the validity of submaximal methods to estimate $\dot{V}O_{2\max}$ and lower-body 1RM in operational firefighters, relative to direct maximal methods and in relation to FFST performance.

## Methods

A total of 22 fully trained full-time operational firefighters (three female) from the North Yorkshire Fire and Rescue Service (NYFRS) volunteered to participate in the study, with physical characteristics for males, females and the overall participant group presented in Table 1. All participants completed written, informed consent and health screening before starting the study, which was approved by the institutional ethics review committee (application number: 2020-3432) in accordance with the Declaration of Helsinki. All participants had previous experience of the physical tests required by (conducted in) this study as part of the annual fitness assessment and/or physical training schedule within NYFRS, including lower-body strength training.

**Table 1.** Mean ± SD physical characteristics for males, females and the overall participant group

	Male (*n* = 19)	Female (*n* = 3)	All (*n* = 22)
Age (years)	34.1 ± 7.9	30.7 ± 7.6	33.6 ± 7.8
Mass (kg)	90.3 ± 11.8	66.3 ± 2.1^*^	87.0 ± 13.5
Height (cm)	182.4 ± 5.4	171.0 ± 5.3^*^	180.9 ± 6.6
Body mass index (kg/m^2^)	27.1 ± 3.1	22.7 ± 1.2^*^	26.5 ± 3.3

^*^Significantly different from men (*P* < 0.05).

The first visit required all participants to first complete the CST followed by a maximal incremental treadmill test, each separated by a rest period of at least 30 minutes. The CST protocol followed that of Sykes [20], with heart rate (Polar H10, Polar, Finland) recorded at the end of each 2-minute stage. Estimated $\dot{V}O_{2\max}$ was established from heart rate using the CST computerized prediction software [20,21]. Maximal incremental testing was performed on a motorized treadmill (Gym Gear T98, UK), with a 3-minute warm-up completed at 5.6 km·h^–1^, followed by a 0.6 km·h^–1^ increase in speed and a 1% increase in gradient every 2 minutes thereafter. These increments continued until a speed of 11.2 km·h^–1^ was achieved, after which the gradient was increased by 2% per minute until volitional exhaustion. Breath-by-breath oxygen uptake was recorded via a metabolic cart (Cortex Metalyzer 3B, Germany) with heart rate recorded using an integrated chest transmitter belt (Polar H10, Polar, Finland). All cardiorespiratory data were time averaged as per the recommendations of Robergs *et al.* [22], whereby $\dot{V}O_{2\max}$ and maximum heart rate (HR_max_) were calculated as the highest 30 seconds average value recorded.

A sub-sample of 10 participants (one female) also completed submaximal and maximal lower-limb strength testing during their first visit, having had a 1-hour recovery period following treadmill testing. The physical characteristics of males, females and the overall sub-group are presented in Table 2. Strength testing adopted a standardized incremental back-squat protocol [18] with an instructor-led mobilization warm-up using a 20-kg Olympic barbell (Gym Gear Ltd, UK). A FLEX (Kinetic Performance Technologies, Australia) laser-optic device was fitted to the right-hand side of the barbell to calculate the movement displacement of the barbell during each lift. A series of progressive loads were then completed based on the estimated 1RM value provided by the FLEX device via the FLEX Stronger app (Kinetic, Australia; firmware version: A714) with standardized encouragement and rest between lifts [23]. Following the submaximal protocol prescribed by the FLEX Stronger app (i.e. 5+ sets, 1–3 reps, between 50–85% of estimated 1RM, with 2-minute recovery between), an estimated 1RM value was calculated by the software and recorded. The submaximal 1RM estimate was not revealed to participants until they had completed the subsequent maximal strength testing protocol. This required the completion of progressively heavier single lifts until failure, with each lift interspersed with a 2-minute recovery (and with two attempts given to lift a failed weight). As such, 1RM was defined as the maximum load (kg) successfully lifted for a full repetition [24]. The first visit concluded with an FFST demonstration to re-familiarize participants with the test.

**Table 2.** Mean ± SD physical characteristics for males, females and the overall sub-group

	Male (*n* = 9)	Female (*n* = 1)	All (*n* = 10)
Age (years)	32.7 ± 6.1	39	33.3 ± 5.8
Mass (kg)	93.0 ± 12.4	68	90.5 ± 14.1
Height (cm)	180.9 ± 5.5	169	179.7 ± 6.4
Body mass index (kg/m^2^)	28.4 ± 3.0	23.8	27.9 ± 3.2

The second visit required participants to complete the FFST to the best of their ability on a flat 25 m course, as per the standardized approach of Siddall *et al*. [25]. The FFST requires the completion of job-specific tasks (i.e. equipment carry, casualty evacuation, hose run) across a timed 1025 m shuttle-run circuit, in the shortest possible overall time. Participants wore full-firefighting PPE (i.e. tunic, salopettes, boots, gloves and helmet), and standardized verbal feedback was provided [6]. Heart rate was recorded throughout (Polar H10, Polar, Finland), with HR_peak_ calculated as the highest 30-second average value recorded during the test [6,22].

Data analysis was completed using SPSS for Windows (Version 28), with means and standard deviations calculated for all variables and normal distribution confirmed by Shapiro–Wilk tests. Differences between estimated and directly measured $\dot{V}O_{2\max}$ and 1RM values were assessed via paired *t*-tests, with Bland–Altman plots [26] used to assess within-subject variation in estimated versus directly measured values, with 95% limits of agreement (LoA) calculated. Pearson’s Product–Moment correlation coefficients established the strength of associations between estimated and directly measured values for $\dot{V}O_{2\max}$ and 1RM and between FFST performance and the estimated and directly measured values for both $\dot{V}O_{2\max}$ and 1RM.

Stepwise multiple regression tests were used to establish predictors of performance in the FFST test. The modelling strategy focussed on separate analyses of those parameters associated with submaximal testing or those associated with maximal testing. Durbin–Watson values <1 or >3 were considered as a violation of statistical assumptions [27]. The prediction model(s) with the highest proportion of explained variance (*R*^2^) and the lowest standard error of the estimate (SEE) were then selected. Non-standardized beta correlation coefficients were used to construct prediction equations for FFST completion time.

Given the potential effect of biological sex on each variable and the relatively small number of females within the study sample, all analyses were performed with and without female participant data. If the removal of female data substantially changed the outcome of any analysis, then this was reported separately from the outcomes for whole group data. For all analyses, statistical significance was set at an alpha value of *P* < 0.05.

## Results

**Figure 1.**
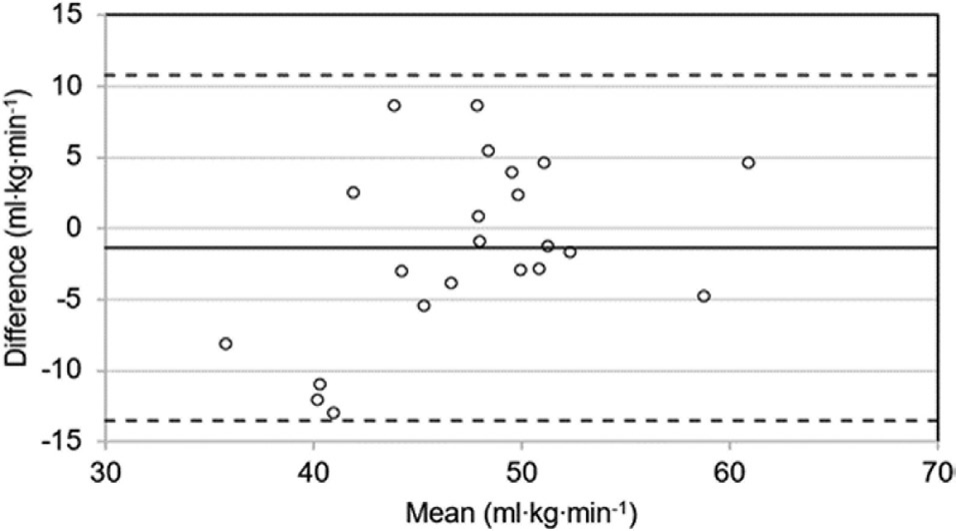
Bland–Altman plot of predicted (CST) versus directly measured VO_2max_. Solid (black) line indicates mean bias (–1.4 ml·kg^–1^·min^–1^) and dashed lines indicate 95% limits of agreement (upper 10.8 ml·kg^–1^·min^–1^, lower –13.5 ml·kg^–1^·min^–1^).

$\dot{V}O_{2\max}$ , HR_max_ and FFST performance data for males, females and the overall participant group are summarized in Table 3. There was a significant moderate positive correlation (*r* = 0.61, *P* < 0.01) between estimated and directly measured $\dot{V}O_{2\max}$ values, which were not significantly different from one another. Bland–Altman analysis established the mean bias by which the CST underestimated directly measured $\dot{V}O_{2\max}$ was 1.4 ml·kg^–1^·min^–1^ (~3%) with a 95% confidence interval for LoA of ±12.2 ml·kg^–1^·min^–1^ (Figure 1).

**Table 3.** Mean ± SD FFST performance, $\dot{V}O_{2\max}$ and HR_max_ values for males, females and the overall participant group

	Male (*n* = 19)	Female (*n* = 3)	All (*n* = 22)
FFST time (s)	585.6 ± 42.1	632.3 ± 47.6	592.0 ± 44.8
FFST HR_peak_ (beats·min^–1^)	181 ± 6	186 ± 2^*^	182 ± 6
FFST HR_peak_ (%HR_max_)	96 ± 2	96 ± 3	97 ± 2
$\dot{V}O_{2\max}$ (ml·kg^–1^·min^–1^)	48.0 ± 5.6	50.1 ± 2.7	48.3 ± 5.3
$\dot{V}O_{2\max}$ (CST) (ml·kg^–1^·min^–1^)	46.7 ± 8.3	47.9 ± 3.4	46.9 ± 7.8
$\dot{V}O_{2\max}$ (L·min^–1^)	4.3 ± 0.6	3.3 ± 0.3	4.2 ± 0.6
HR_max_ (beats·min^–1^)	189 ± 8	194 ± 6	190 ± 8
HR_max_ (Tanaka) (beats·min^–1^)	184 ± 6	187 ± 5	184 ± 5

^*^Significantly different from men (*P* < 0.05).

**Figure 2.**
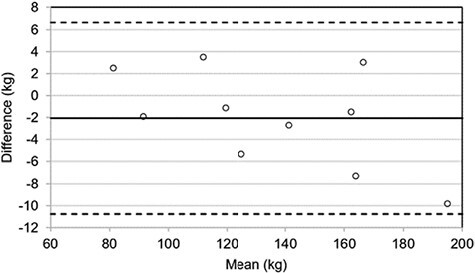
Bland–Altman plot of estimated versus directly measured 1RM. The solid (black) line indicates mean bias (–2.1 kg) and dashed lines indicate 95% limits of agreement (upper 6.6 kg, lower –10.7 kg).

1RM data for males, females and the overall participant group are summarized in Table 4. A strong positive correlation (*r* = 0.995, *P* < 0.01) was observed between estimated and directly measured back-squat 1RM, with no significant difference between these values. Bland–Altman analysis revealed the mean bias by which the submaximal method underestimated directly measured 1RM was 2.1 kg (~2%), with a 95% confidence interval for LoA of ±8.7 kg (Figure 2).

**Table 4.** Mean ± SD estimated and directly measured 1RM values for males, females and the overall sub-group

	Male (*n* = 9)	Female (*n* = 1)	All (*n* = 10)
Squat 1RM (kg)	141.2 ± 35.3	92.5	136.3 ± 34.8
Squat 1RM (FLEX) (kg)	139.1 ± 32.5	90.6	134.2 ± 32.5
Squat 1RM (1RM/body mass)	1.5 ± 0.3	1.4	1.5 ± 0.3
Squat 1RM (FLEX) (1RM/body mass)	1.5 ± 0.3	1.3	1.5 ± 0.3

One repetition maximum (1RM), submaximal 1RM estimate (FLEX).

FFST performance of all participants was 592 ± 45 seconds, with an HR_peak_ of 182 ± 6 beats·min^–1^ (97 ± 2 % HR_max_) observed. Both estimated and directly measured values for $\dot{V}O_{2\max}$ demonstrated a weak negative correlation with FFST performance (*r* = –0.467 and –0.379, respectively). The removal of female data substantially increased the strength of relationship between directly measured $\dot{V}O_{2\max}$ and FFST performance (*r* = –0.561). For all sub-group data, weak negative correlations were observed for estimated and directly measured 1RM values versus FFST performance (*r *= –0.388 and –0.320, respectively).

The strongest overall prediction models for FFST performance included age, body mass index (BMI), and direct maximal measures of 1RM and $\dot{V}O_{2\max}$ (L·min^–1^) irrespective of female data being included (*R* = 0.764, *R*^2^  = 0.583, SEE = 45.2 seconds) or removed (*R* = 0.891, *R*^2^ = 0.587, SEE = 27. 9 seconds) from the analysis. As such, the following prediction equations of FFST performance time were produced for male-only and mixed-sex data:


$$\begin{array}{l} \begin{array}{llll} & FFST\;completion\;time\;\left(seconds\right)\; & \left(male\;only\;data\right)\;=\;537.101\left(2.096\times Age\right)\; & \left(0.380\times 1RM\right)\left(62.841\times VO_{2max}\right)+(16.033\times BMI)\; \end{array} \end{array}$$

$$\begin{array}{l} \begin{array}{llll} & FFST\;completion\;time\left(seconds\right)\; & \left(mixed\;\text{-}\;sex\;data\right)=\;748.432\left(2.666\times Age\right)\; & \left(0.407\times 1RM\right)\left(90.867\times VO_{2max}\right)+(14.189\times BMI)\; \end{array} \end{array}$$

When based on only main group data (i.e. without 1RM values), the strongest prediction model for FFST performance included age, BMI and directly measured $\dot{V}O_{2\max}$ (L·min^–1^) irrespective of female data being included (*R* = 0.657, *R*^2^ = 0.432, SEE = 36.5 seconds) or removed (*R* = 0.682, *R*^2^ = 0.465, SEE = 35.9 seconds) from the analysis.

When based on only submaximal data, the strongest prediction model for FFST performance included age, BMI, estimated $\dot{V}O_{2\max}$ and estimated 1RM, irrespective of female data being included (*R* = 0.592, *R*^2^ = 0.350, SEE = 56.4 seconds) or removed (*R* = 0.805, *R*^2^ = 0.648, SEE = 36.5 seconds) from the analysis.

## Discussion

This study has shown that the CST underestimates $\dot{V}O_{2\max}$ by 1.4 ml·kg^–1^·min^–1^ (~3%) in full-time operational firefighters compared to direct maximal treadmill assessment, with a margin of error (i.e. 95% confidence interval for LoA) of ±12.16 ml·kg^–1^·min^–1^. Greater mean differences have been reported across a number of studies examining submaximal $\dot{V}O_{2\max}$ estimate methods in physically demanding occupations, including firefighting, with mean error values of between 2.9 and 7.0 ml·kg^–1^·min^–1^ being accepted for tests to be deemed suitably valid for use [28–31]. More specifically, Dolezal *et al*. [32] reported greater mean error (~11%) in $\dot{V}O_{2\max}$ estimates derived for firefighters from submaximal treadmill testing than the current study, supporting the relative validity of the CST. Further to these points, we found a significant positive correlation and no statistically significant differences between estimated and directly measured $\dot{V}O_{2\max}$ values. There was also a significant negative correlation between estimated $\dot{V}O_{2\max}$ values derived from the CST and FFST performance time.

These findings suggest that the CST has sufficient validity to provide a relatively low-cost estimate of $\dot{V}O_{2\max}$ which translates well to role-specific task performance of firefighters, but that practitioners within the fire service should use and interpret the CST with a degree of caution, particularly when making operational decisions on borderline cases. In cases where individuals fall within the LoA, it is advised that a more accurate follow-up test is used to clarify results, such as maximal breath-by-breath testing [32]. However, given that a relatively large proportion of firefighters may fall into these LoA for estimated $\dot{V}O_{2\max}$, the need to deploy more sophisticated tests across a substantial number of firefighters is likely to outweigh any savings that the initial use of the CST currently offers. There may, therefore, be a need for firefighter-specific $\dot{V}O_{2\max}$ prediction equations to be developed to translate CST data more effectively (i.e. estimate $\dot{V}O_{2\max}$ with greater accuracy). As highlighted by Dolezal *et al*. [32], this may require a critical evaluation of age-predicted HR_max_ calculations used as part of the CST [33], given the inherent error of using generalized equations in populations differing from the original research context.

This study has also demonstrated that estimates of back-squat 1RM derived using the FLEX laser-optic device are significantly correlated to, and not significantly different from, directly measured back-squat 1RM values. Whilst this novel testing method appears to underestimate 1RM by ~2% compared to direct maximal assessment, with a margin of error (i.e. 95% confidence interval for LoA) of ±8.7 kg (~6%), this level of accuracy would appear to compare favourably to the CST and aforementioned studies of submaximal $\dot{V}O_{2\max}$ estimate protocols. Whilst there is limited evidence from physically demanding occupation settings, such agreement would appear to sit favourably with that reported by studies examining submaximal velocity-based estimation of back-squat 1RM [34]. Furthermore, both estimated and directly measured back-squat 1RM were included in the strongest models for predicting FFST performance from submaximal and direct maximal methods, respectively. This is in keeping with the work of Michaelides *et al*. [35], which established that strength measures are able to explain a significant proportion of variation in firefighter ability test performance. The current findings, therefore, add further support to the importance of strength and more specifically to the testing and training of lower-body strength in operational firefighters [4,13,14]. Whilst practitioners should adopt a degree of caution interpreting estimated 1RM values, this novel approach to submaximal strength testing may offer sufficient validity to profile firefighter fitness effectively, without exposure to levels of fatigue associated with direct maximal testing.

A key strength of this study is that it is the first, to our knowledge, to directly examine the validity of the CST in a group of operational UK firefighters which allows the findings to be generalized to this population. Whilst CST reliability in firefighters has been examined [7,36], it is important for the population-specific validity of any submaximal estimate test to be established [37]. Given the widespread use of this test within the fire service, and firefighter research, this study offers important population-specific insight into the use and interpretation of CST results obtained from firefighters. Another key strength is that this study has established a novel lower-body submaximal strength test that has good levels of firefighter-specific validity. The submaximal FLEX strength test is relativity cheap, portable and accessible, and therefore has the potential to be easily adopted across fire and rescue services.

Whilst the sample size of the current study is equivalent or greater than previous similar research (e.g. [38]), this equated to ~8% of the total pool of full-time firefighters operating in the NYFRS and so the findings would have been enhanced by a larger, more representative, sample. More specifically, it would appear that the current study group was younger and more aerobically fit than the typical (i.e. service mean) profile of NYFRS firefighter and also relative to previous firefighter studies (i.e. >40 years and <48 ml·kg^–1^·min^–1^) [5,6]. It is, therefore, important for future research to establish how older (i.e. middle-aged) firefighters with lower fitness levels respond to each of the tests examined in the current study. Whilst the proportion of females in the study (~13%) was relatively higher than that seen currently within the NYFRS (~8%), and falls within the range used in previous research to establish the minimum fitness standards of firefighters (i.e. 7–19%) [5,6], the inclusion of more females in future research work (and from a broader range of fire and rescue services) would enhance our understanding of fitness testing and development in physically demanding occupations such as firefighting.

A further limitation of this study was the relatively narrow range of submaximal test protocols examined. In terms of aerobic capacity estimation, the current study did not consider the relative validity of the Chester Treadmill/Walk Test, for example. Similarly, strength testing was limited to only the back-squat movement, so there would be value in establishing the relative validity of submaximal 1RM estimates across a range of upper and lower body lifts that may be relevant to the demands of firefighting (e.g. Overhead press, deadlift) [4,12,14]. Finally, whilst the reliability of the laser-optic device technology and associated maximal strength estimates used in the current study have been reported previously [18], there is a lack of firefighter-specific evidence relating to this. Given that relative strength levels may influence the reliability of estimated 1RM derived using this method [24], future studies should examine its population-specific reliability for both male and female firefighters.
